# *Shigella flexneri* with Ciprofloxacin Resistance and Reduced Azithromycin Susceptibility, Canada, 2015

**DOI:** 10.3201/eid2211.160883

**Published:** 2016-11

**Authors:** Christiane Gaudreau, Pierre A. Pilon, Gilbert Cornut, Xavier Marchand-Senecal, Sadjia Bekal

**Affiliations:** Centre Hospitalier de l’Université de Montréal–Hôpital Saint-Luc, Montreal, Quebec, Canada (C. Gaudreau, G. Cornut, X. Marchand-Senecal);; Université de Montréal, Montreal (C. Gaudreau, P.A. Pilon, G. Cornut, X. Marchand-Senecal, S. Bekal);; Centre Intégré Universitaire de Santé et de Services Sociaux du Centre-Sud-de-l’île-de-Montreal, Montreal (P.A. Pilon);; Laboratoire de Santé Publique du Québec/Institut National de Santé Publique du Québec, Sainte-Anne-de-Bellevue, Quebec (C. Gaudreau, S. Bekal)

**Keywords:** *Shigella* spp., emergence, azithromycin, drug resistance, ciprofloxacin, Quebec, homosexual, men who have sex with men, bacteria

**To the Editor:** In 2015, a locally acquired, multidrug-resistant *Shigella flexneri* infection was identified in Montreal, Quebec, Canada, in an HIV-positive man who had sex with men (MSM). In September, the 53-year-old man consulted his physician at an outpatient clinic after experiencing abdominal pain, fatigue, and diarrhea without blood in stools or fever. The week before the symptom onset, although he had not traveled, he had unprotected oral and anal sexual contact in a Montreal bathhouse with a man visiting Canada from an unknown country. The patient did not work in daycare centers or healthcare facilities, and he was not a food handler. He did not have sex during illness.

He was HIV positive and was receiving antiretroviral treatment; recent CD4 cell count was 480 × 10^6^/L, and HIV viral load was <40 copies/mL. *S. flexneri* was isolated from his culture of a fecal sample, and *Neisseria gonorrhoeae,* diagnosed by PCR, was found in a throat specimen. The patient did not have a medical record of other past sexually transmitted infections. 

Phenotypic identification of the *S. flexneri* was confirmed at Laboratoire de Santé Publique du Québec (*1*). Serologic identification, pulsed-field gel electrophoresis (PFGE), and antimicrobial susceptibility testing were performed as described (*1*). This *S. flexneri,* serotype 2a pulsovar 21 (a new PFGE combination pattern in the province of Quebec), was resistant to ampicillin, trimethoprim/sulfamethoxazole (TMP/SMX), nalidixic acid, ciprofloxacin, tetracycline, and chloramphenicol. The isolate was also nonsusceptible to azithromycin and amoxicillin/-clavulanic acid, and susceptible to ceftriaxone, cefixime, ertapenem, and gentamicin (Table). The *mph*(A) gene, which codes for the macrolide 2′-phosphotransferase, tested positive by PCR (*1*).

**Table:** Antimicrobial susceptibility of the *Shigella flexneri*,serotype 2a pulsovar 21, isolated in Montreal, Quebec, Canada, 2015*

Antimicrobial agent	Disk diffusion, mm	MIC, mg/L	Interpretation
Ampicillin	6	≥32	R
TMP/SMX	6	≥320	R
Ciprofloxacin	12	≥4 and 8	R
Nalidixic acid	6	NA	R
Ceftriaxone	33	≤0.25	S
Cefixime	26	0.25	S
Azithromycin†	6	>256	NS
Tetracycline	6	32	R
Chloramphenicol	NA	>256	R
Amoxicillin-clavulanic acid	14	16	I
Ertapenem	NA	≤0.5	S
Gentamicin	21	≤1	S
*I, intermediate; NA, not available; NS, nonsusceptible; R, resistant; S, susceptible; TMP/SMX, trimethoprim/sulfamethoxazole. †Azithromycin epidemiologic cutoff values for wild-type (MIC ≤8 mg/L) and non–wild-type (MIC ≥16 mg/L) *Shigella flexneri* (*2*) and the susceptibility and resistance breakpoints for the other 11 antimicrobial agents were CLSI *Enterobacteriaceae *breakpoints (*2*).			

On day 10 of diarrhea, the patient was treated with ceftriaxone, 250-mg dose, intramuscularly, followed by cefixime, 800 mg/day, for 5 days; the patient’s condition showed progressive improvement. Two control cultures of fecal specimens were negative 7 and 16 days, respectively, after completion of a regimen of cefixime.

*Shigella* spp. are transmitted from person-to-person through low inocula of the bacteria, directly or indirectly (*1**,**3**–**5*). In MSM, *Shigella* spp. are mostly transmitted sexually, with clusters documented in many countries (*1**,**3**–**5*). In Canada and the United States, *Shigella* isolates have high levels of resistance to ampicillin and TMP/SMX (*1**,**3**–**6*). In adult patients, when antimicrobial drug treatment is indicated, ciprofloxacin and azithromycin are, respectively, the agents of first and second choices for treating *Shigella* infections (*1**,**3**–**5*). 

In the United States, *Shigella* spp. resistant to at least nalidixic acid and azithromycin have been found in surveillance isolates: 1/293 in 2011 (*Shigella* spp.), 1/353 in 2012 (*S. sonnei*), and 1 of 344 in 2013 (*S. flexneri*) (*6*). In Illinois and Montana, during September 2014–April 2015, 3 of 5 patients infected with multidrug-resistant *S. sonnei* (resistant to ampicillin, TMP/SMX, ciprofloxacin, and nalidixic acid and nonsusceptible to azithromycin), identified themselves as MSM, and 2 of these patients had diarrhea for >14 days (*3*).

Clinical treatment failure has been reported in patients infected with azithromycin-nonsusceptible *Shigella* isolates treated with this drug (*7**,**8*), including 1 of our patients (unpub. data). In a previous study, the *mph*(A) gene was acquired by 4 of 7 locally acquired *Shigella* pulse types infecting MSM. This raises concern that reduced *Shigella* susceptibility to azithromycin is developing rapidly (*1*). Azithromycin epidemiologic cutoff values for wild- and non–wild-types of *S. flexneri* and *S. sonnei* are newly reported by CLSI (*8*). In recent years, ciprofloxacin-resistant and/or azithromycin-nonsusceptible *Shigella* spp. acquired during international travel or acquired locally were reported in the United States and in our hospital center (*1,3–6*; unpub. data). *S. flexneri* that is resistant to ceftriaxone and ciprofloxacin has been reported in the United States (*9*). Infections with multidrug-resistant *Shigella* spp. may be of longer duration and have higher costs (*3*).

When evaluating patients with diarrhea, physicians should identify risk factors and request bacterial cultures of fecal specimens. Antimicrobial drug susceptibility testing of *Shigella* isolates is essential for effective antimicrobial drug treatment. Serologic identification and PFGE are essential for epidemiologic purposes for ascertaining clusters or multidrug-resistant *Shigella* isolates (*1**,**3**–**5*). Patients with *Shigella* infection should be advised about preventive practices such as frequent handwashing and precautions when handling food and water (*3*). MSM should use barriers during oral, anal, and genital sex and wash their genitals, anus, and hands before and after sex (*1**,**3**–**5*).

We suggest obtaining 2 control cultures of fecal specimens on days 2 and 3 after the patient completes antimicrobial treatment for infection with multidrug-resistant *Shigella* spp. Patients should avoid sex during symptomatic infections and wait for 2 negative stool cultures. Montreal public health officials investigated and counselled this patient as they do for every patient with *Shigella* infections. In Quebec, physicians and microbiology laboratories are notified of *Shigella* clusters and multidrug-resistant *Shigella* infections.

To our knowledge, no other ciprofloxacin-resistant and azithromycin-nonsusceptible *Shigella flexneri* isolates have been documented in the province of Quebec. No PFGE matches to *S. flexneri* serotype 2a pulsovar 21 have been identified in Canada. Multidrug-resistant *Shigella* isolates, including those with both resistance to ciprofloxacin and nonsusceptibility to azithromycin, may be underestimated and incidence may be increasing (*1**,**3**–**5*).
